# 

**Published:** 1894-09

**Authors:** 


ESTABLISHED 1855. SUBSCRIPTION, $2.00.

LIBRARY Index S. G. O.

### ATLANTA MEDICAL AND SURGICAL JOURNAL.

OLD SERIES, VOL. XXVI. NEW SERIES, VOL. XI. } ATLANTA, GA., SEPTEMBER, 1894. No. 7.

LUTHER B. GRANDY, M.D., MANAGING EDITOR.

M. B. HUTCHINS, M.D., BUSINESS MANAGER.

